# Prevalence of ultrasound-detected knee synovial abnormalities in a middle-aged and older general population—the Xiangya Osteoarthritis Study

**DOI:** 10.1186/s13075-021-02539-2

**Published:** 2021-06-02

**Authors:** Ting Jiang, Tuo Yang, Weiya Zhang, Michael Doherty, Yuqing Zhang, Jie Wei, Aliya Sarmanova, Michelle Hall, Zidan Yang, Jiatian Li, Gwen S. Fernandes, Abasiama D. Obotiba, Sameer A. Gohir, Philip Courtney, Chao Zeng, Guanghua Lei

**Affiliations:** 1 Department of Orthopaedics, Xiangya Hospital, Central South University, 87 Xiangya Road, Changsha, 410008 Hunan China; 2 Department of Ultrasonography, Xiangya Hospital, Central South University, Changsha, China; 3 Division of Rheumatology, Orthopaedics and Dermatology, University of Nottingham, Nottingham, UK; 4 Pain Centre Versus Arthritis UK, Nottingham, UK; 5 Health Management Center, Xiangya Hospital, Central South University, Changsha, China; 6 Division of Rheumatology, Allergy, and Immunology, Department of Medicine, Massachusetts General Hospital, Harvard Medical School, Boston, USA; 7 The Mongan Institute, Massachusetts General Hospital, Harvard Medical School, Boston, USA; 8 Hunan Key Laboratory of Joint Degeneration and Injury, Xiangya Hospital, Central South University, Changsha, China; 9 Musculoskeletal Research Unit, Bristol Medical School, University of Bristol, Bristol, UK; 10 School of Health Sciences, University of Nottingham, Nottingham, UK; 11 NIHR Nottingham Biomedical Research Centre, School of Medicine, University of Nottingham, Nottingham, UK; 12 Population Health Sciences, Bristol Medical School, University of Bristol, Bristol, UK; 13 Department of Rheumatology, Nottingham University Hospitals NHS Trust, Nottingham, UK; 14 National Clinical Research Center for Geriatric Disorders, Xiangya Hospital, Central South University, Changsha, China

**Keywords:** Synovial abnormalities, Knee, Ultrasonography, General population

## Abstract

**Background:**

There is paucity of data on the prevalence of ultrasound-detected synovial abnormalities in the general population, and the relationship between synovial changes and knee pain remains unclear. We examined the prevalence of synovial abnormalities on ultrasound and the relationship of these features with knee pain and radiographic osteoarthritis (ROA) in a community sample.

**Methods:**

Participants aged 50 years or over were from the Xiangya Osteoarthritis Study, a community-based cohort study. Participants were questioned about chronic knee pain and underwent (1) ultrasonography of both knees to determine presence of synovial hypertrophy (≥ 4 mm), effusion (≥ 4 mm), and Power Doppler signal [PDS; yes/no]; and (2) standard radiographs of both knees (tibiofemoral and patellofemoral views) to determine ROA.

**Results:**

There were 3755 participants (mean age 64.4 years; women 57.4%). The prevalence of synovial hypertrophy, effusion, and PDS were 18.1% (men 20.2%; women 16.5%), 46.6% (men 49.9%; women 44.2%), and 4.9% (men 4.9%; women 5.0%), respectively, and increased with age (P for trend < 0.05). Synovial abnormalities were associated with knee pain, with adjusted odds ratios (aORs) of 2.39 (95% confidence interval [CI] 2.00–2.86) for synovial hypertrophy, 1.58 (95%CI 1.39–1.80) for effusion, and 4.36 (95%CI 3.09–6.17) for PDS. Similar associations with ROA were observed, the corresponding aORs being 4.03 (95%CI 3.38–4.82), 2.01 (95%CI 1.76–2.29), and 6.49 (95%CI 4.51–9.35), respectively. The associations between synovial hypertrophy and effusion with knee pain were more pronounced among knees with ROA than those without ROA, and the corresponding *P* for interaction were 0.004 and 0.067, respectively.

**Conclusions:**

Knee synovial hypertrophy and effusion are more common and increase with age, affecting men more than women. All three ultrasound-detected synovial abnormalities associate both with knee pain and ROA, and knee synovial hypertrophy or effusion and ROA may interact to increase the risk of knee pain.

**Supplementary Information:**

The online version contains supplementary material available at 10.1186/s13075-021-02539-2.

## Background

Knee pain is highly prevalent in the middle-aged and older population, with a prevalence ranging from 25.0 to 47.1% [1–3]. Chronic knee pain decreases lower limb strength [4], limits daily activities [5], reduces quality of life [6], and associates with increased all-cause mortality [7]. Although commonly associated with knee osteoarthritis (OA), the pathogenesis of this common disorder has not been fully elucidated, though synovial pathology is thought to play an important role and is, therefore, a common treatment target [8, 9]. Magnetic resonance imaging (MRI) especially contrast-enhanced MRI (CE-MRI) has been used to assess knee synovial abnormalities [8, 10, 11]. However, owing to its limited availability, long examination time, high associated costs, possible allergic reactions, and potential risk of contrast-induced nephrogenic systemic fibrosis [12], it is unfeasible to routinely perform MRI or CE-MRI as an initial and subsequently repeated test, especially for multiple joint assessments.

Ultrasonography is an accurate, widely available, inexpensive and noninvasive imaging modality that has been used increasingly for evaluating synovial abnormalities [13, 14]. Without requirement of contrast agents to visualize synovium, ultrasound offers more accurate assessment of synovial abnormalities than conventional clinical examination [15] and can be as sensitive and specific as CE-MRI [16]. In addition, Power Doppler (PD) ultrasound modes provide a unique opportunity to detect indirect signs of increased vascularization, which reflects with high sensitivity joint inflammatory activity [17]. However, there are few epidemiological studies that describe the prevalence of knee synovial abnormalities on ultrasound or examine the relation of these features to knee symptoms and radiographic osteoarthritis (ROA) in the general population [18–22]. Lack of such data makes it difficult to interpret the diagnostic information provided by ultrasound in people who have symptoms that are possibly attributable to synovial abnormalities. In particular, over half of people with knee pain have no ROA [23, 24], and in clinical practice, it is unclear how to investigate and manage such people and whether additional imaging with ultrasound would be of clinical value.

To help fill this gap, we conducted a population-based study to determine the prevalence of ultrasound-detected synovial abnormalities (synovial hypertrophy, effusion, and PD signal [PDS]) of knee joints and examined their associations with knee pain and ROA among middle-aged and older men and women who were representative of the general population living at Longshan County, China.

## Methods

### Study design and population

Participants in this cross-sectional study were from the Xiangya Osteoarthritis Study (XO Study), an ongoing community-based longitudinal study of the natural history of OA and its risk factors in Longshan County, Hunan Province, China [25, 26]. Based on a multistage, stratified random sampling of villages, all residents aged 50 years or older from 25 randomly selected villages were invited. This study was approved by the Ethics Committee of Xiangya Hospital, Central South University (201510506) and registered on ClinicalTrials.gov (NCT04033757). Written informed consent was obtained from all participants.

Among 4742 randomly selected individuals, 4080 (86.04%) consented to participate at baseline. The XO Study consists of 3 sub-cohorts. Sub-cohort 1 was recruited in 2015 when 1469 individuals completed their interviews and clinical examinations [25]. Of these 1207 and 1181 participants attended the Year 1 (Year 2016) and Year 2 (Year 2017) follow-ups, respectively. Sub-cohorts 2 (*n* = 1271) and 3 (*n* = 1340) were recruited in 2018 and 2019, respectively. Bilateral knee ultrasound was taken for each Sub-cohort 1 participant in 2017 (i.e., the second year follow-up visit), and for Sub-cohort 2 participants in 2018 and Sub-cohort 3 in 2019 (i.e., both baseline examinations) (Supplemental Figure 1). Among 3792 participants who attended for assessment, 37 (1.0%) were excluded from the current analyses because of not undergoing ultrasound examination (*n* = 32), previous knee replacement surgery (*n* = 1), current severe knee injury (*n* = 1), artificial limb (*n* = 1), or severe lower limb deformity (*n* = 2). Thus, the final sample comprised 3755 individuals.

### Assessment of ultrasound

One trained sonographer (TJ, over 10 years’ experience in musculoskeletal ultrasonography) performed all ultrasound examinations using a Philips CX30 ultrasound machine with a 4–12-MHz linear transducer. A pulse repetition frequency of 400 Hz was used for PD examination, the gain being adjusted until the background signal was removed. The sonographer was blinded to the participant’s knee pain status and radiographic score.

Both knees were assessed with the participant supine and the knee in 30° flexion. The suprapatellar bursa was scanned according to the Outcome Measures in Rheumatology (OMERACT) atlas [14]. Synovial hypertrophy and effusion were assessed using OMERACT-7 definitions [27] (Supplemental Figure 2). The maximal synovial thickness and effusion depth were measured in millimeters using the longitudinal axis. Presence of synovial hypertrophy was defined as synovial thickness ≥ 4 mm, and presence of joint effusion was defined as depth of effusion ≥ 4 mm according to the European League Against Rheumatism (EULAR) study [28]. PDS observed in the synovial membrane in both longitudinal and transverse planes was scored using a semi-quantitative grading system, from 0 to 3 (0 = absent, 1 = mild, 2 = moderate, 3 = marked or severe) [29] (Supplemental Figure 3). Participants were defined as having synovial hypertrophy, effusion, or PDS if these features were present in either knee.

To assess intra-observer reliability of the ultrasound measures, 30 grey scale and 30 PD stored ultrasound images from 55 participants with different severity of synovial abnormalities were selected, and then re-read by the same assessor (TJ) at a minimum of 12 weeks after acquisition of ultrasound, blind of the original scores. For inter-observer reliability, two assessors (TJ, MH) independently assessed the same 30 grey scale and 30 PD ultrasound images. Weighted kappa statistic was used for semi-quantitative data and intra-class correlation coefficient (ICC) was used for continuous data.

### Assessment of pain

All the visit participants were asked about knee symptoms with the question: “During the past 12 months, have you had pain, aching, or stiffness in or around your right or left knee on most days for at least 1 month?” A positive response was considered to indicate presence of chronic knee pain. The severity of current knee pain was assessed by the Western Ontario and McMaster Universities Osteoarthritis Index (WOMAC), with a 5-point pain scale from 0 to 4 [30]. We divided pain severity into three categories: no pain (WOMAC score 0 on all five pain questions), mild to moderate pain (WOMAC score 1/2 on any of the pain questions), and severe to extreme pain (WOMAC score 3/4 on any of the pain questions) [31].

### Assessment of ROA

Participants underwent a bilateral weight-bearing posterior-anterior semi-flexed tibiofemoral (TF) view radiograph, as well as skyline patellofemoral (PF) views with the person in a supine position and the knee flexed to 45° using a wedge for accuracy. Radiographs of the TF and PF joints were graded using Kellgren-Lawrence (KL) grades (range 0–4) (Supplemental Figure 4 and 5). Knee ROA was defined as presence of a KL grade ≥ 2 in any TF or PF joint of each knee.

A single researcher (primary reader, TY, orthopedic surgeon) scored all knee films. Prior to the assessment, one senior bone-and-joint radiologist (Piran Aliabadi) scored twenty films which were selected from the XO Study. The primary reader reviewed the twenty pre-scored films to calibrate his reading with the senior radiologist’s reading. Preliminary reading of batches of randomly selected films continued until the primary reader reached a high level of intra- and inter-rater agreement. With each new batch of films (*n* = 50), we commingled two films from the pre-scored films by the senior radiologist (Piran Aliabadi), and five previously read radiographs by the primary reader himself to test the reliability.

### Assessment of covariates

Demographic characteristics were collected face-to-face by trained health professional interviewers using standard questionnaires. Age, sex, smoking, alcohol drinking, education, and knee injury history were recorded. Height and weight were measured, and body mass index (BMI) was calculated as weight (kg) divided by square of height (m^2^). We grouped BMI into three categories: normal (BMI < 25 kg/m^2^), overweight (BMI 25–29.9 kg/m^2^), and obesity (BMI ≥ 30 kg/m^2^).

### Statistical analysis

Continuous variables were expressed as mean ± standard deviation (SD), while categorical variables were expressed as percentage. We calculated the sex- and age-specific (50–59, 60–69, ≥ 70 years) prevalence of each ultrasound-detected synovial feature as the number of cases was divided by the total number of participants in each stratum separately and its 95% confidence intervals [32]. We examined the associations of each knee synovial feature with knee pain and ROA. The odds ratio (OR) and related 95% confidence interval (CI) were generated from the Generalized Estimating Equations using the PROC GENMOD procedure in SAS with logit links, with adjustment for potential confounders (age, sex, BMI [< 25, ≥ 25 kg/m^2^], smoking status, alcohol consumption, education level, and knee injury history).

As pain is a subjective experience and unique to each person, we further conducted a within-person knee-matched analysis by examining the relationship of knee synovial abnormalities to knee pain among participants who had discordant pain status among two knees [31]. This approach eliminated person-level confounders [31]. Participants with chronic knee pain in one knee but not the other were eligible for the analysis. In addition, participants with bilateral knee pain who reported discordance between knees in categories of pain severity were selected to examine the relationship between synovial abnormalities and knee pain severity. Conditional logistic regression was used to examine associations of synovial abnormalities with each pain measurement because of the matched nature of the data.

To assess whether the association between each synovial abnormality and prevalent knee pain varied according to knee ROA status, we grouped knees into four categories according to the presence or absence of knee-specific synovial abnormalities and ROA and examined relation of each category to knee pain. Using interaction analyses, we tested whether the association between each synovial abnormalities and knee pain was modified by knee ROA status.

All *P* values were 2-sided and *P* < 0.05 was considered significant. All statistical analyses were conducted using SAS V.9.4 (SAS Institute, Cary, NC, USA).

## Results

Of the 3755 participants included in this analysis, 57.4% were women, the mean age was 64.4 (SD 9.2) years, and the average BMI was 24.0 kg/m^2^ (Table 1). Prevalence of chronic knee pain was 33.2%, and prevalence of knee ROA (TF/PF joint OA) was 37.0%. The intra- and inter-rater reliability were excellent for synovial hypertrophy (range of ICCs 0.94–0.99), effusion (range of ICCs 0.96–0.98), PDS (range of weighted Kappa statistics 0.82–1.00), and KL grade (range of weighted Kappa statistics 0.76–0.91) (Supplemental Table 1).

**Table 1:** Characteristics of the study sample

	Total (***n*** = 3755)
**Women, n (%)**	2157 (57.4%)
**Age, years (%)**	
50–59	33.7
60–69	36.1
≥ 70	30.2
**Height, cm (mean ± SD)**	151.1 ± 8.0
**Weight, kg (mean ± SD)**	54.8 ± 9.9
**BMI, kg/m**^**2**^ **(mean ± SD)**	24.0 ± 3.6
**Overweight, n (%)**^a^	1099 (30.4%)
**Obesity, n (%)**^b^	215 (5.9%)
**Smoking status (%)**	
Non-smoker	65.4
Ex-smoker	4.4
Current smoker	30.2
**Alcohol drinking (%)**	
Non-drinker	53.8
Ex-drinker	11.5
Current drinker	34.7
**Education (educated, %)**^c^	67.2
**Knee injury history (%)**^d^	3.4
**Chronic knee pain (%)**^e^	33.2
**Knee radiographic OA (%)**^f^	37.0

*n* number, *SD* standard deviation, *BMI* body mass index, *OA* osteoarthritis

^a^Overweight was defined as a BMI of 25 to 29.9 kg/m^2^. In total, 3621 participants had complete anthropometric measurements (weight and height)

^b^Obesity was defined as a BMI of ≥ 30 kg/m^2^

^c^Educated was defined as primary school or above

^d^Knee injury history was defined as history of knee injury severely restricting walking for at least 1 week

^e^Chronic knee pain was based on a self-report of whether participant had pain, aching, or stiffness in or around their right or left knee on most days for at least 1 month within the past year

^f^Knee radiographic OA was confirmed if at least one knee (tibiofemoral or patellofemoral joint) was rated as Kellgren and Lawrence grade ≥ 2

**Fig. 1:**
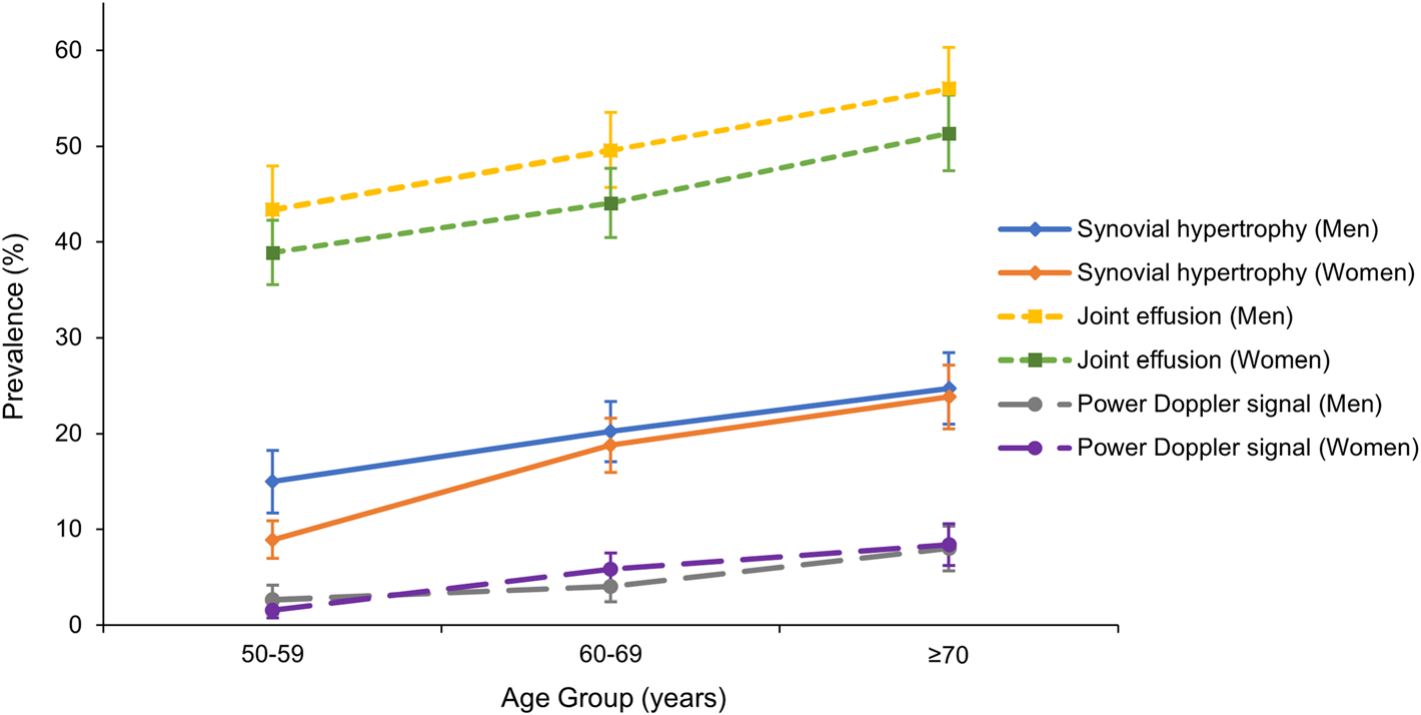
Prevalence of knee synovial abnormalities on ultrasound among middle-aged and elderly persons, according to age group and sex. The error bars denote 95% confidence intervals

The prevalence of synovial hypertrophy, effusion, and PDS were 18.1% (men 20.2%; women 16.5%), 46.6% (men 49.9%; women 44.2%), and 4.9% (men 4.9%; women 5.0%), respectively. As shown in Fig. 1 and Supplemental Table 2, the prevalence of each specific synovial feature increased with age (*P* for trend < 0.05). Men had higher prevalence of synovial hypertrophy and effusion than women (*P* < 0.01), but there was no sex difference in the prevalence of PDS.

The associations of synovial abnormalities with knee pain severity are shown in Table 2**.** Compared with knees without synovial hypertrophy, crude ORs of mild to moderate pain versus no pain was 1.81 (95%CI 1.51–2.16) and severe to extreme pain versus no pain was 4.04 (95%CI 3.18–5.13), and the results persisted after adjusting for potential confounders. The unadjusted ORs of knee pain were 2.54 (95%CI 2.15–2.99) for synovial hypertrophy, 1.61 (95%CI 1.43–1.82) for effusion, and 4.98 (95%CI 3.63–6.82) for PDS, respectively (Supplemental Table 3). Results from within-person knee-matched analyses of synovial abnormalities among participants with discordant knee pain status were similar (Supplemental Table 4).

**Table 2:** Association of knee synovial abnormalities on ultrasound and knee pain severity

Synovial abnormalities	Knee pain severity^b^		
	No pain	Mild to moderate pain	Severe to extreme pain
**Synovial hypertrophy**			
No, n (%)	4145 (63.6)	1937 (29.7)	439 (6.7)
Yes, n (%)	360 (44.0)	304 (37.2)	154 (18.8)
Crude OR (95% CI)	1.00 (reference)	1.81 (1.51, 2.16)	4.04 (3.18, 5.13)
Adjusted OR (95% CI)^a^	1.00 (reference)	1.79 (1.48, 2.15)	3.67 (2.82, 4.78)
**Joint effusion**			
No, n (%)	3192 (64.6)	1426 (28.9)	323 (6.5)
Yes, n (%)	1313 (54.7)	815 (34.0)	270 (11.3)
Crude OR (95% CI)	1.00 (reference)	1.39 (1.23, 1.57)	2.03 (1.66, 2.48)
Adjusted OR (95% CI)^a^	1.00 (reference)	1.41 (1.24, 1.60)	1.93 (1.55, 2.39)
**Power Doppler signal**			
No, n (%)	4446 (62.4)	2156 (30.2)	530 (7.4)
Yes, n (%)	59 (28.5)	85 (41.1)	63 (30.4)
Crude OR (95% CI)	1.00 (reference)	2.97 (2.09, 4.23)	8.96 (5.99, 13.39)
Adjusted OR (95% CI)^a^	1.00 (reference)	2.77 (1.92, 4.01)	7.19 (4.50, 11.48)

*n* number, *OR* odds ratio, *CI* confidence interval

^a^Adjusted for age, sex, BMI, smoking status, alcohol consumption, education, and knee injury history

^b^Severity of knee pain was categorized into severe to extreme pain (WOMAC score of 3 or 4 on any of the five pain questions), mild to moderate pain (WOMAC score of 1 or 2 on any of the five pain questions), and no pain (WOMAC score of 0 on all five pain questions)

As shown in Table 3, compared with knees without synovial hypertrophy, knees with hypertrophy had 3.04 (95%CI 2.52–3.67), 6.20 (95%CI 4.90–7.83), and 10.22 (95%CI 7.40–14.11) times higher odds of KL grades 2, 3, and 4, respectively. Findings for the relationship between effusion and PDS with severity of knee ROA were similar. These relationships remained after adjustment for confounders. The crude ORs of knee ROA were 4.27 (95%CI 3.63–5.02) for synovial hypertrophy, 2.01 (95%CI 1.79–2.26) for effusion, and 7.97 (95%CI 5.72–11.08) for PDS (Supplemental Table 5).

**Table 3:** Association of knee synovial abnormalities on ultrasound and Kellgren-Lawrence grades

Synovial abnormalities	Kellgren-Lawrence grades			
	< 2	2	3	4
**Synovial hypertrophy**				
No, n (%)	4979 (74.9)	1179 (17.7)	363 (5.5)	125 (1.9)
Yes, n (%)	343 (41.2)	247 (29.6)	155 (18.6)	88 (10.6)
Crude OR (95% CI)	1.00 (reference)	3.04 (2.52, 3.67)	6.20 (4.90, 7.83)	10.22 (7.40, 14.11)
Adjusted OR (95% CI)^a^	1.00 (reference)	2.98 (2.43, 3.65)	6.07 (4.69, 7.86)	9.69 (6.82, 13.76)
**Joint effusion**				
No, n (%)	3829 (76.0)	855 (17.0)	261 (5.2)	94 (1.8)
Yes, n (%)	1493 (61.2)	571 (23.4)	257 (10.5)	119 (4.9)
Crude OR (95% CI)	1.00 (reference)	1.71 (1.50, 1.96)	2.53 (2.08, 3.07)	3.25 (2.36, 4.46)
Adjusted OR (95% CI)^a^	1.00 (reference)	1.75 (1.51, 2.03)	2.53 (2.04, 3.14)	3.15 (2.23, 4.44)
**Power Doppler signal**				
No, n (%)	5269 (72.5)	1372 (18.9)	456 (6.3)	169 (2.3)
Yes, n (%)	53 (24.9)	54 (25.3)	62 (29.1)	44 (20.7)
Crude OR (95% CI)^a^	1.00 (reference)	3.91 (2.64, 5.80)	13.52 (9.07, 20.15)	25.88 (16.34, 40.99)
Adjusted OR (95% CI)^a^	1.00 (reference)	3.46 (2.27, 5.29)	11.64 (7.45, 18.19)	20.93 (12.30, 35.61)

*n* number, *OR* odds ratio, *CI* confidence interval

^a^Adjusted for age, sex, BMI, smoking status, alcohol consumption, education level, and knee injury

According to Table 4, the relation to knee pain was stronger among knees with both synovial hypertrophy and ROA (crude OR = 5.51, 95%CI 4.27–7.11) than knees with ROA alone (crude OR = 2.45, 95%CI 2.10–2.87) or knees with synovial hypertrophy alone (crude OR = 1.46, 95%CI 1.10–1.93) (*P* for interaction = 0.004). A similar pattern was also observed when association between effusion and knee pain was examined, but not between PDS and knee pain. Adjusting for potential confounders (Table 4) and conducting a within-person knee-matched analysis among participants with discordant knee pain status (Supplemental Table 6) did not change the results substantially.

**Table 4:** Association of knee pain stratified by knee synovial abnormalities on ultrasound and radiographic osteoarthritis status

Synovial abnormalities and ROA status	No. of patients with knee pain (%)	Crude OR (95% CI)^a^	Adjusted OR (95% CI)^a^	***P*** value of interaction
**Synovial hypertrophy**				0.004
Neither synovial hypertrophy nor ROA	1013 (20.6)	1.00 (reference)	1.00 (reference)	
Synovial hypertrophy only	86 (25.4)	1.31 (1.00, 1.71)	1.46 (1.10, 1.93)	
ROA only	772 (47.7)	3.51 (3.04, 4.04)	2.45 (2.10, 2.87)	
Synovial hypertrophy and ROA	313 (65.8)	7.40 (5.86, 9.34)	5.51 (4.27, 7.11)	
**Joint effusion**				0.067
Neither joint effusion nor ROA	744 (19.7)	1.00 (reference)	1.00 (reference)	
Joint effusion only	355 (24.1)	1.29 (1.10, 1.52)	1.34 (1.13, 1.59)	
ROA only	560 (47.8)	3.74 (3.17, 4.40)	2.62 (2.18, 3.14)	
Joint effusion and ROA	525 (56.8)	5.35 (4.47, 6.40)	3.86 (3.18, 4.69)	
**Power Doppler signal**				0.794
Neither Power Doppler signal nor ROA	1075 (20.7)	1.00 (reference)	1.00 (reference)	
Power Doppler signal only	24 (45.3)	3.18 (1.81, 5.58)	3.12 (1.70, 5.73)	
ROA only	971 (50.0)	3.84 (3.35, 4.39)	2.69 (2.31, 3.12)	
Power Doppler signal and ROA	114 (74.0)	10.94 (7.34, 16.30)	8.34 (5.44, 12.80)	

*OR* odds ratio, *CI* confidence interval, *ROA* radiographic osteoarthritis

^a^Adjusted for age, sex, BMI, smoking status, alcohol consumption, education level, and knee injury history

## Discussion

In this large general population-based study, nearly half the participants (46.6%) had knee effusion on ultrasound and synovial hypertrophy and PDS were found in 18.1% and 4.9% of participants, respectively. The prevalence of synovial abnormalities increased with age, and men appeared to have higher prevalence of synovial hypertrophy and effusion, but not PDS, than women. Synovial abnormalities associated with both knee pain and ROA. The association with knee pain was independent from ROA, and the presence of synovial abnormality (synovial hypertrophy or joint effusion) and ROA interacted to increase the risk of knee pain.

Few studies have reported the prevalence of ultrasound-detected synovial abnormalities in community-based samples who were not selected on the basis of knee problems [19–22]. In Newcastle in the UK, effusion was detected in 24% and 20% of right and left knees, respectively, among 311 participants aged 61 to 63 years [19]. In the Italian Bruneck population-based cohort, the prevalence of synovial hypertrophy, effusion, and PDS were 43.9%, 60.4%, and 22.6%, respectively, among 433 survivors in the fourth follow-up (927 participants at baseline) [20]. In the Nottingham Knee Pain and Related Health in the Community Study in the UK, the prevalence was 23.9% for synovial hypertrophy, 50.9% for effusion, and 0.7% for PDS in 163 participants with no knee pain or ROA [21]. In the Johnston County Osteoarthritis Project, evidence of effusion/synovitis on ultrasound was found in 80.3% of 396 knees of 203 participants using a semi-quantitative assessment atlas [22]. The prevalence of synovial abnormalities in our study was comparable with these previous studies, suggesting similar findings in diverse populations (China, USA, UK, and Italy). Several previous studies also reported higher prevalence of synovial hypertrophy and effusion in men than women [20, 21, 33]. This finding may be partly explained by the fact that men normally have thicker synovium and more synovial fluid than women [21]. The high prevalence of abnormal ultrasound values in this and previous general population samples suggest that, rather than using a fixed cut-off of ≥ 4 mm for “abnormal” synovial hypertrophy and effusion, different cut-offs for presence of hypertrophy and effusion, and for men and women, might be calculated from normal ranges and incorporated into future studies.

Previous studies have sought an association between ultrasound-detected synovial changes and knee pain, but results have been inconclusive [20, 28, 34–48]. Most studies were conducted in people with knee OA [28, 34–47] and were conditional on an intermediate variable (i.e., requirement of ROA), which may introduce selection bias (i.e., collider bias [49]) that dilutes the effect estimate. Our study provided empirical evidence that presence of synovial hypertrophy, effusion, and PDS all strongly associated both with presence and severity of knee pain, independent of potential confounders. We also demonstrated a positive association between synovial abnormalities and presence and severity of ROA. These results accord with previous findings [22, 48, 50], and reinforce that synovial changes are involved in knee OA.

Our study involved a large, general population-based sample (*n* = 3755) and had sufficient power to generate a representative estimate of prevalence of three synovial abnormalities in the general population, and to examine each of these features in relation to knee pain and ROA. Secondly, when examining the association between synovial abnormalities and knee pain, we compared the two knees within people who had knees discordant for presence of knee pain and pain severity. Being that within an individual all person-level factors that influence pain would contribute equally to both knees, this approach eliminated confounders between individuals and allowed determination of valid effect estimates of specific synovial abnormalities on knee pain. Thirdly, all ultrasound examinations were performed by a single experienced musculoskeletal sonographer, thus eliminating inter-observer variability. Nevertheless, we assessed inter-observer reliability with another expert musculoskeletal ultrasonographer and the results were excellent, providing external validation of the measures.

Our study has some limitations. First, participants were residents in rural areas of China with low prevalence of obesity (5.6%); thus, caution must be taken when generalizing our results to urban, suburban, or obese populations. Second, we used EULAR recommended cut-offs for synovial abnormalities, which may not be generalizable to the Chinese population. Third, we were unable to confirm a diagnosis of rheumatoid arthritis based on the 1987 American College of Rheumatology (ACR) or 2010 American College of Rheumatology/European League Against Rheumatism (ACR/EULAR) criteria in the current study. Nevertheless, when subjects who self-reported rheumatoid arthritis with typical radiographic evidence or anti-rheumatoid therapy [51] were excluded, the results did not change materially (data not shown). Fourth, the associations between synovial hypertrophy and effusion, but not PDS, with knee pain were more pronounced among knees with ROA than those without ROA. We postulate that the low prevalence of PDS in the current study may have limited the study power when interaction of PDS and OA on knee pain was assessed. Fifth, this is a cross-sectional study, so we cannot confirm temporal association and causality between synovial abnormalities, ROA, and pain. The association identified between synovial features and ROA does not necessarily mean that synovial features are the causes or consequences of the ROA. In addition, the associations between synovial abnormalities and knee pain remained similar after further adjusting for knee ROA (data not shown), indicating that these associations may be independent of ROA. However, since previous studies report that synovial abnormalities increased the risk of incident knee ROA [52, 53], adjusting for ROA may be akin to adjusting for an intermediate variable between synovial abnormalities and knee pain, leading to a biased effect estimate [49].

Since ultrasound enables real-time noninvasive imaging of synovial abnormalities at low cost, knee ultrasound especially for initial assessment of knee synovial abnormalities could be encouraged. In addition, knee synovial abnormalities have been found to associate with knee pain and ROA. Thus, the high prevalence of synovial abnormalities in the general population in our study suggests a potentially higher burden of knee pain and OA than previously thought. However, whether these synovial changes are beneficial or damaging remains unclear. They may reflect the joint’s attempted repair response to insult, which is characterized by increased physiological/pathological activity and hyperplasia of joint tissues. Indeed, a modest degree of inflammation as part of repair, rather than aggressive synovitis, is supported by the lower prevalence of PDS. Furthermore, approximately half of the participants with knee pain but no ROA had synovial abnormalities. However, longitudinal studies are needed to verify whether ultrasound-detected synovial abnormalities could predict incident knee ROA. Considering that synovial abnormalities present a potentially modifiable pathological process [9], additional imaging with ultrasound may be of value in clinical practice.

## Conclusions

In conclusion, knee synovial hypertrophy and effusion are more common and increase with age, affecting men more than women. All three ultrasound-detected synovial abnormalities associate both with knee pain and ROA, and knee synovial hypertrophy or effusion and ROA may interact to increase the risk of knee pain.

## Supplementary Information

**Additional file 1: Supplemental Figure 1.** Recruitment and enrollment of study participants in Xiangya Osteoarthritis Study (XO Study). **Supplemental Figure 2.** Longitudinal ultrasonographic scan of suprapatellar recess (B mode). SH, synovial hypertrophy; E, joint effusion. **Supplemental Figure 3.** Longitudinal ultrasonographic scan of suprapatellar recess (Power Doppler mode). Power Doppler signal (PDS) at synovial membrane was scored using a semi-quantitative grading system, from 0 to 3 (0=absent, 1=mild, 2=moderate, 3=marked or severe). (A) grade 0, (B) grade 1, (C) grade 2. We did not detected grade 3 PDS in knee joints of participants of our study. **Supplemental Figure 4.** Bilateral weight-bearing posterior-anterior tibio-femoral radiographs, assessed according to Kellgren and Lawrence (KL) criteria: (A) grade 0, (B) grade 1, (C) grade 2, (D) grade 3 and (E) grade 4. **Supplemental Figure 5.** Skyline views of patellofemoral radiographs, assessed according to Kellgren and Lawrence (KL) criteria: (A) grade 0, (B) grade 1, (C) grade 2, (D) grade 3 and (E) grade 4. **Supplemental Table 1.** The intra- and inter-rater reliability for ultrasound-detected synovial abnormalities and Kellgren-Lawrence grade of knee radiography. **Supplemental Table 2.** Prevalence of knee synovial abnormalities on ultrasound among middle-aged and elderly persons, according to age group and sex. **Supplemental Table 3.** Association of knee synovial abnormalities on ultrasound and knee pain. **Supplemental Table 4.** Association of knee synovial abnormalities on ultrasound and knee pain among people with two knees discordant for knee pain. **Supplemental Table 5.** Association of knee synovial abnormalities on ultrasound and knee radiographic osteoarthritis. **Supplemental Table 6.** Association of knee pain stratified by knee synovial abnormalities on ultrasound and radiographic osteoarthritis status among people with two knees discordant for knee pain.

## Data Availability

The datasets analyzed during the current study are available from the corresponding authors on reasonable request.

## Abbreviations

ROA: Radiographic osteoarthritis
PDS: Power Doppler signal
aORs: Adjusted odds ratios
CI: Confidence interval
OA: Osteoarthritis
MRI: Magnetic resonance imaging
CE-MRI: Contrast-enhanced MRI
PD: Power Doppler
XO Study: Xiangya Osteoarthritis Study
OMERACT: Outcome Measures in Rheumatology
EULAR: European League Against Rheumatism
ICC: Intra-class correlation coefficient
WOMAC: Western Ontario and McMaster Universities Osteoarthritis Index
TF: Tibiofemoral
PF: Patellofemoral
KL: Kellgren-Lawrence
BMI: Body mass index
SD: Standard deviation
OR: Odds ratio
