# Supplementary material for: Prevalence of ultrasound-detected knee synovial abnormalities in a middle-aged and older general population—the Xiangya Osteoarthritis Study
Source: Arthritis Res Ther. 2021 Jun 2;23:156. doi: 10.1186/s13075-021-02539-2 (PMC8170794; doi:10.1186/s13075-021-02539-2)
Supplement: Supplementary file 1 — Additional file 1: Supplemental Figure 1. Recruitment and enrollment of study participants in Xiangya Osteoarthritis Study (XO Study). Supplemental Figure 2. Longitudinal ultrasonographic scan of suprapatellar recess (B mode). SH, synovial hypertrophy; E, joint effusion. Supplemental Figure 3. Longitudinal ultrasonographic scan of suprapatellar recess (Power Doppler mode). Power Doppler signal (PDS) at synovial membrane was scored using a semi-quantitative grading system, from 0 to 3 (0=absent, 1=mild, 2=moderate, 3=marked or severe). (A) grade 0, (B) grade 1, (C) grade 2. We did not detected grade 3 PDS in knee joints of participants of our study. Supplemental Figure 4. Bilateral weight-bearing posterior-anterior tibio-femoral radiographs, assessed according to Kellgren and Lawrence (KL) criteria: (A) grade 0, (B) grade 1, (C) grade 2, (D) grade 3 and (E) grade 4. Supplemental Figure 5. Skyline views of patellofemoral radiographs, assessed according to Kellgren and Lawrence (KL) criteria: (A) grade 0, (B) grade 1, (C) grade 2, (D) grade 3 and (E) grade 4. Supplemental Table 1. The intra- and inter-rater reliability for ultrasound-detected synovial abnormalities and Kellgren-Lawrence grade of knee radiography. Supplemental Table 2. Prevalence of knee synovial abnormalities on ultrasound among middle-aged and elderly persons, according to age group and sex. Supplemental Table 3. Association of knee synovial abnormalities on ultrasound and knee pain. Supplemental Table 4. Association of knee synovial abnormalities on ultrasound and knee pain among people with two knees discordant for knee pain. Supplemental Table 5. Association of knee synovial abnormalities on ultrasound and knee radiographic osteoarthritis. Supplemental Table 6. Association of knee pain stratified by knee synovial abnormalities on ultrasound and radiographic osteoarthritis status among people with two knees discordant for knee pain. [file 13075_2021_2539_MOESM1_ESM.docx]

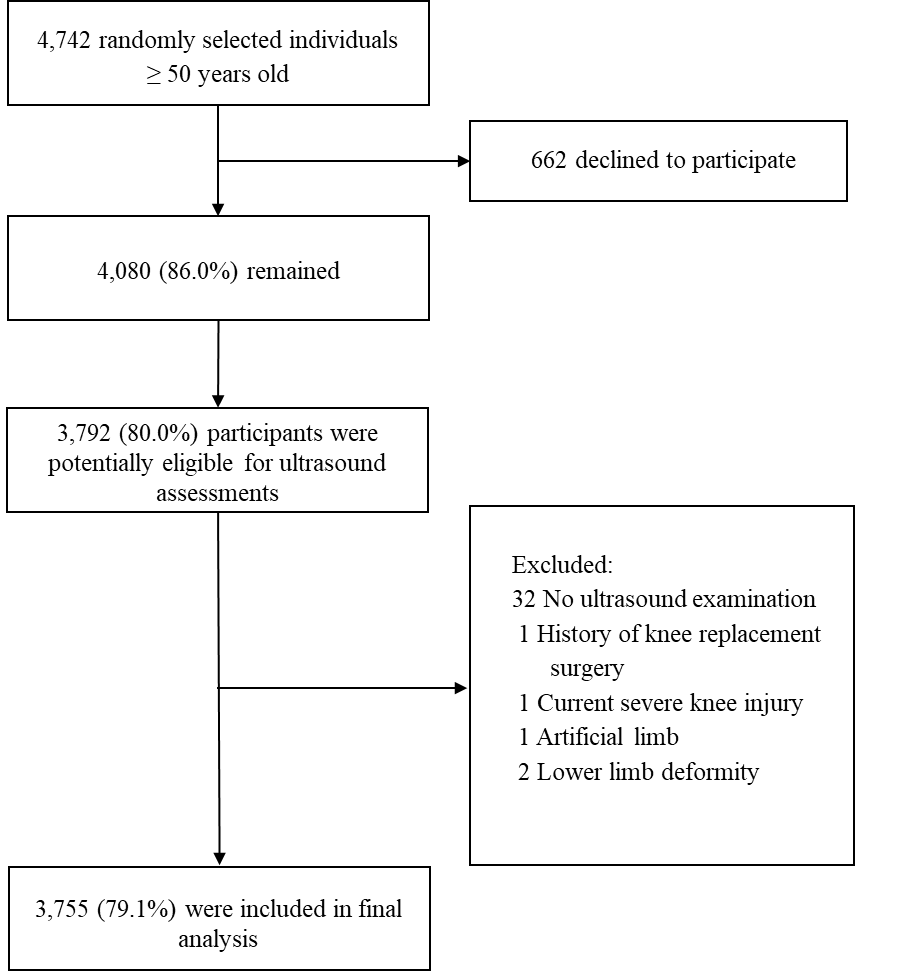


**Supplemental Figure 1. Recruitment and enrollment of study participants in Xiangya Osteoarthritis Study (XO Study).**


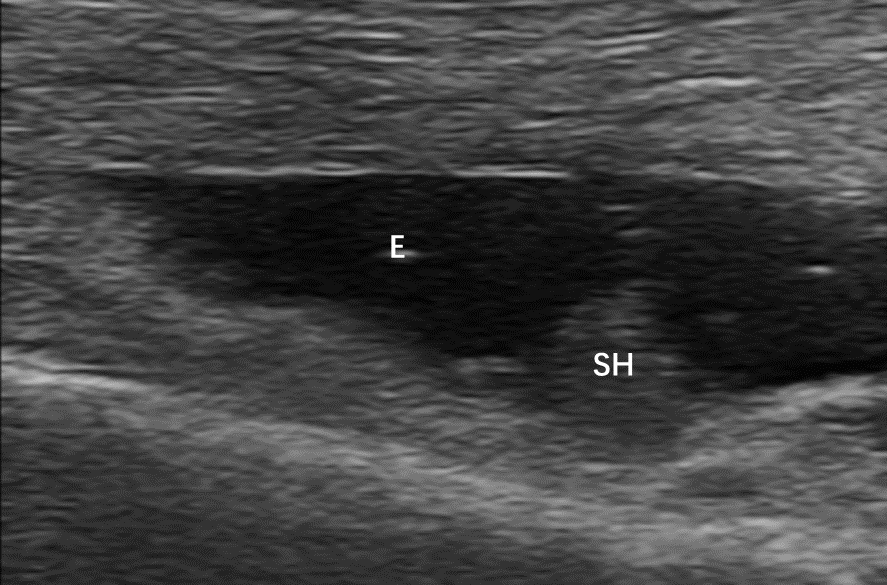


**Supplemental Figure 2. Longitudinal ultrasonographic scan of suprapatellar recess (B mode).** SH, synovial hypertrophy; E, joint effusion.


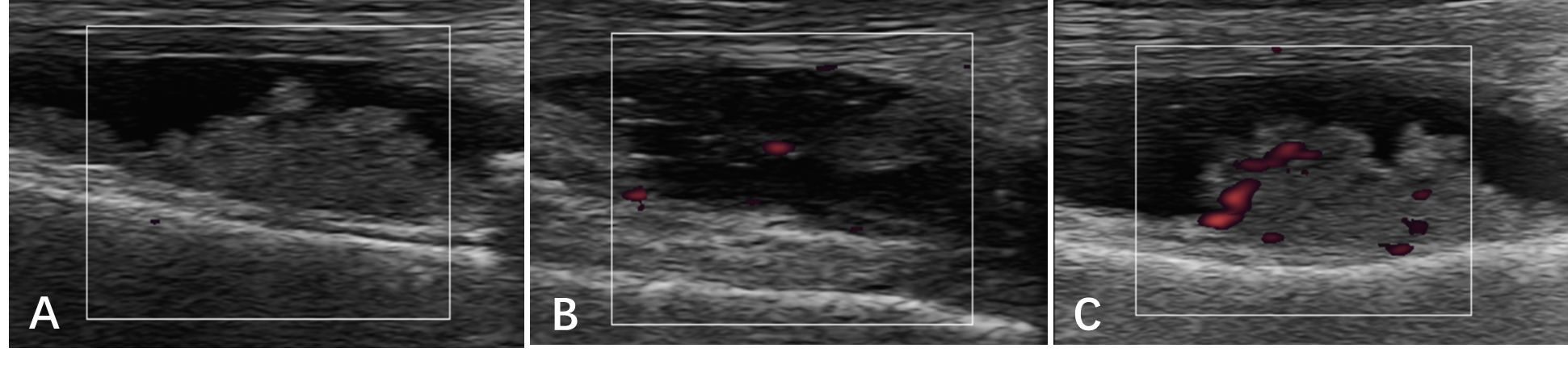


**Supplemental Figure 3. Longitudinal ultrasonographic scan of suprapatellar recess (Power Doppler mode).** Power Doppler signal (PDS) at synovial membrane was scored using a semi-quantitative grading system, from 0 to 3 (0=absent, 1=mild, 2=moderate, 3=marked or severe). (A) grade 0, (B) grade 1, (C) grade 2. We did not detected grade 3 PDS in knee joints of participants of our study.


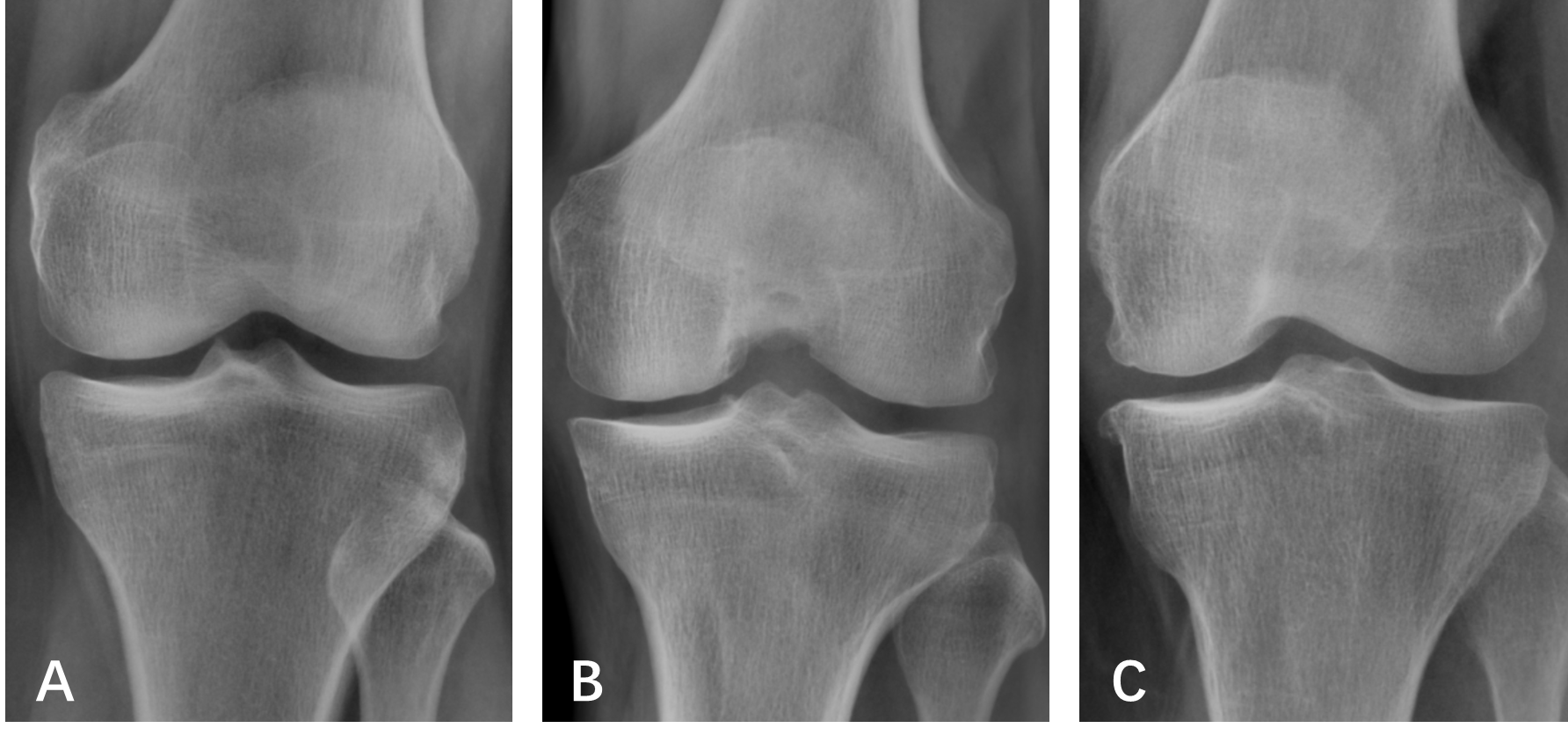


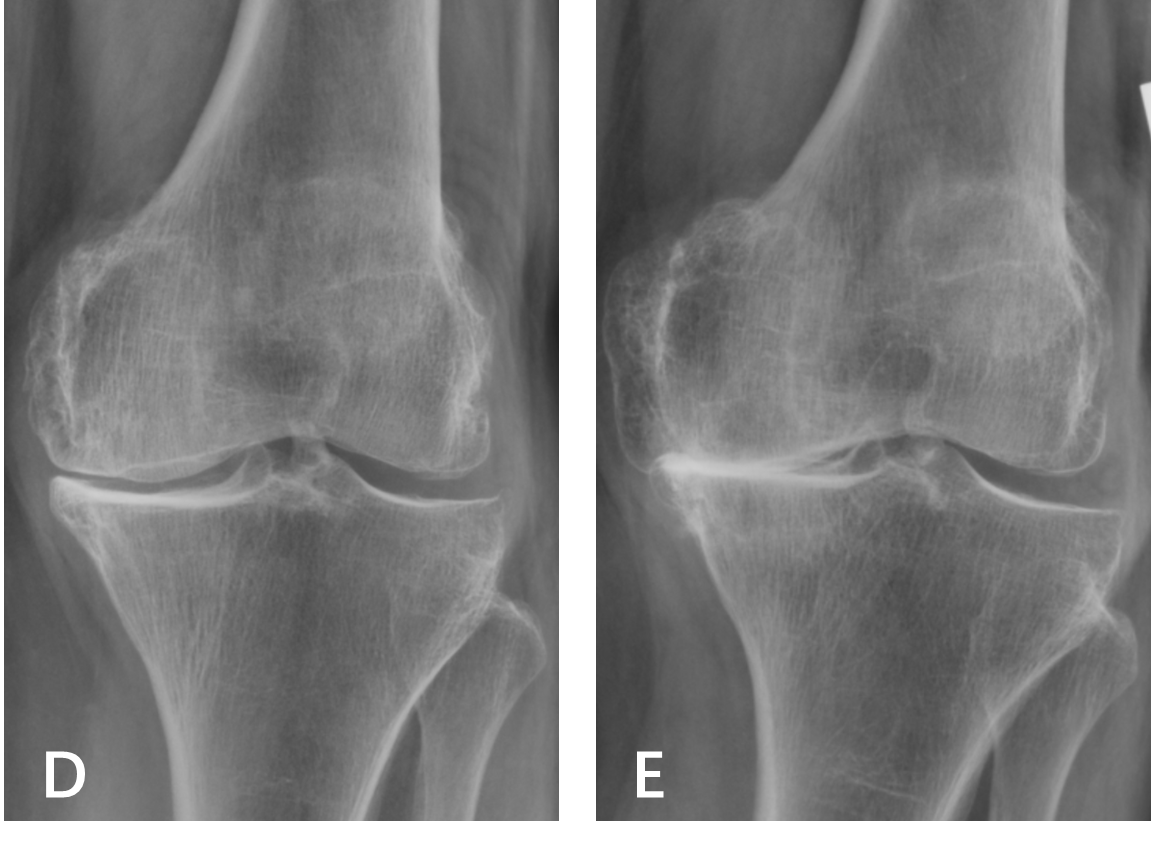


**Supplemental Figure 4. Bilateral weight-bearing posterior-anterior tibio-femoral radiographs, assessed according to Kellgren and Lawrence (KL) criteria: (A) grade 0, (B) grade 1, (C) grade 2, (D) grade 3 and (E) grade 4.**


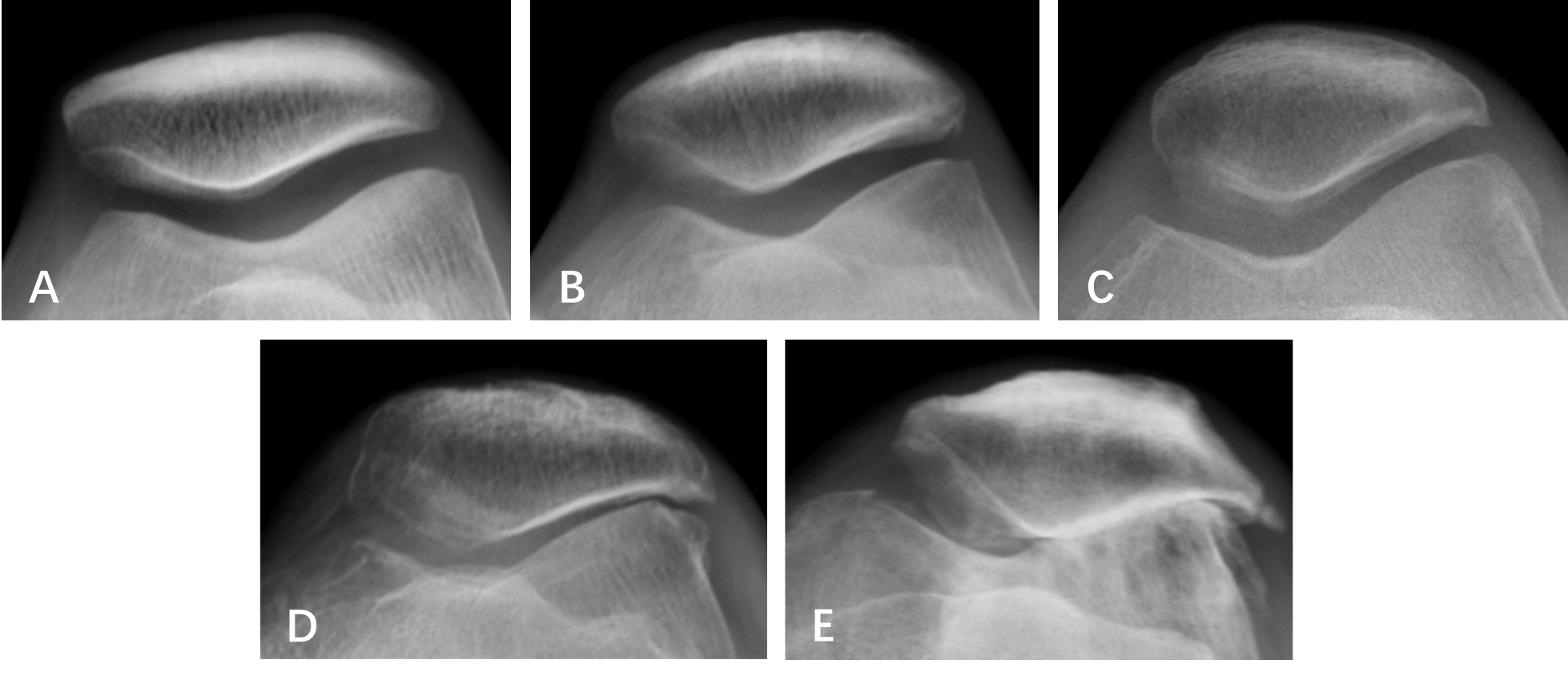


**Supplemental Figure 5. Skyline views of patellofemoral radiographs, assessed according to Kellgren and Lawrence (KL) criteria: (A) grade 0, (B) grade 1, (C) grade 2, (D) grade 3 and (E) grade 4.**

**Supplemental Table 1. The intra- and inter-rater reliability for ultrasound-detected synovial abnormalities and Kellgren-Lawrence grade of knee radiography**

|  |  | **Intra-rater reliability** | **Inter-rater reliability** |
| --- | --- | --- | --- |
| **Ultrasonography** | Synovial hypertrophy,  ICC (95% CI) | 0.99 (0.98-1.00) | 0.94 (0.87-0.97) |
|  | Synovial effusion,  ICC (95% CI) | 0.98 (0.92-0.99) | 0.96 (0.93-0.98) |
|  | Power Doppler Signal,  Weighted-Kappa (95% CI) | 1.00(1.00-1.00) | 0.82 (0.66-0.97) |
|  |  |  |  |
| **Radiography** | Kellgren-Lawrence grade,  Weighted-Kappa (95% CI) | 0.91 (0.88-0.95) | 0.76 (0.62-0.91) |
| ICC, intra-class correlation coefficient; CI, confidence interval. | | | |

**Supplemental Table 2. Prevalence of knee synovial abnormalities on ultrasound among middle-aged and elderly persons, according to age group and sex**

| **Age group (years)** | **Synovial hypertrophy** | **Joint effusion** | **Power Doppler signal** |
| --- | --- | --- | --- |
| 50-59 |  |  |  |
| Men | 15.0 (12.0, 18.6) | 43.4 (38.9, 48.0) | 2.6 (1.5, 4.6) |
| Women | 8.9 (7.1, 11.0) | 38.9 (35.6, 42.3) | 1.6 (0.9, 2.7) |
| 60-69 |  |  |  |
| Men | 20.2 (17.3, 23.5) | 49.6 (45.7, 53.5) | 4.0 (2.7, 5.8) |
| Women | 18.8 (16.1, 21.8) | 44.1 (40.5, 47.7) | 5.8 (4.3, 7.7) |
| ≥70 |  |  |  |
| Men | 24.7 (21.2, 28.6) | 56.0 (51.7, 60.3) | 8.0 (5.9, 10.6) |
| Women | 23.8 (20.6, 27.3) | 51.4 (47.4, 55.3) | 8.4 (6.4, 10.8) |

Data were expressed as prevalence (95% confidence interval).

**Supplemental Table 3.** **Association of knee synovial abnormalities on ultrasound and knee pain**

| **Synovial abnormalities** | **Knee pain** | |
| --- | --- | --- |
|  | **No** | **Yes** |
| **Synovial hypertrophy** |  |  |
| No, n (%) | 4,761 (72.7) | 1,790 (27.3) |
| Yes, n (%) | 420 (51.2) | 401 (48.8) |
| Crude OR (95% CI) | 1.00 (reference) | 2.54 (2.15, 2.99) |
| Adjusted OR (95% CI) * | 1.00 (reference) | 2.39 (2.00, 2.86) |
| **Joint effusion** |  |  |
| No, n (%) | 3,651 (73.6) | 1,307 (26.4) |
| Yes, n (%) | 1,530 (63.4) | 884 (36.6) |
| Crude OR (95% CI) * | 1.00 (reference) | 1.61 (1.43, 1.82) |
| Adjusted OR (95% CI) * | 1.00 (reference) | 1.58 (1.39, 1.80) |
| **Power Doppler signal** |  |  |
| No, n (%) | 5,112 (71.3) | 2,053 (28.7) |
| Yes, n (%) | 69 (33.3) | 138 (66.7) |
| Crude OR (95% CI) * | 1.00 (reference) | 4.98 (3.63, 6.82) |
| Adjusted OR (95% CI) * | 1.00 (reference) | 4.36 (3.09, 6.17) |

n, number; OR, odds ratio; CI, confidence interval.

* adjusted for age, sex, BMI, smoking status, alcohol consumption, education and knee injury history.

**Supplemental Table 4. Association of knee synovial abnormalities on ultrasound and knee pain** **among people with two knees discordant for knee pain**

|  | **Pairs of knees discordant for knee pain (n=247)** | | **Pairs of knees discordant for knee pain severity (n=323)** | | |
| --- | --- | --- | --- | --- | --- |
|  | **No pain** | **Pain** | **No pain** | **Mild to moderate pain** | **Severe to extreme pain** |
| **Synovial hypertrophy** |  |  |  |  |  |
| OR (95% CI) ^#^ | 1.00 (reference) | 2.47 (1.41, 4.34) | 1.00 (reference) | 1.86 (1.11, 3.12) | 2.75 (0.88, 8.64) |
| **Joint effusion** |  |  |  |  |  |
| OR (95% CI) ^#^ | 1.00 (reference) | 1.96 (1.22, 3.15) | 1.00 (reference) | 1.54 (1.01, 2.35) | 2.20 (0.76, 6.33) |
| **Power Doppler signal** |  |  |  |  |  |
| OR (95% CI) ^#^ | 1.00 (reference) | 4.50 (1.52, 13.30) | 1.00 (reference) | 3.00 (1.09, 8.25) | 3.50 (0.73, 16.85) |

n, number; OR, odds ratio; CI, confidence interval.

# adjusted for knee injury history.

**Supplemental Table 5. Association of knee synovial** **abnormalities on ultrasound and knee radiographic osteoarthritis**

| **Synovial abnormalities** | **Knee radiographic osteoarthritis** | |
| --- | --- | --- |
|  | **No** | **Yes** |
| **Synovial hypertrophy** |  |  |
| No, n (%) | 4,979 (74.9) | 1,667 (25.1) |
| Yes, n (%) | 343 (41.2) | 490 (58.8) |
| Crude OR (95% CI) | 1.00 (reference) | 4.27 (3.63, 5.02) |
| Adjusted OR (95% CI) * | 1.00 (reference) | 4.03 (3.38, 4.82) |
| **Joint effusion** |  |  |
| No, n (%) | 3,829 (76.0) | 1,210 (24.0) |
| Yes, n (%) | 1,493 (61.2) | 947 (38.8) |
| Crude OR (95% CI) | 1.00 (reference) | 2.01 (1.79, 2.26) |
| Adjusted OR (95% CI) * | 1.00 (reference) | 2.01 (1.76, 2.29) |
| **Power Doppler signal** |  |  |
| No, n (%) | 5,269 (72.5) | 1,997 (27.5) |
| Yes, n (%) | 53 (24.9) | 160 (75.1) |
| Crude OR (95% CI) | 1.00 (reference) | 7.97 (5.72, 11.08) |
| Adjusted OR (95% CI) * | 1.00 (reference) | 6.49 (4.51, 9.35) |

n, number; OR, odds ratio; CI, confidence interval.

* adjusted for age, sex, BMI, smoking status, alcohol consumption, education level and knee injury history.

**Supplemental Table 6. Association of knee pain stratified by knee synovial abnormalities on ultrasound and radiographic osteoarthritis status among people with two knees discordant for knee pain**

| **Synovial abnormalities and ROA status** | **No. of knees with pain (%)** | **OR (95% CI) *** | **P value of interaction** |
| --- | --- | --- | --- |
| **Synovial hypertrophy** |  |  | 0.061 |
| Neither synovial hypertrophy nor ROA | 121 (43.8) | 1.00 (reference) |  |
| Synovial hypertrophy only | 12 (44.4) | 1.16 (0.47, 2.68) |  |
| ROA only | 65 (52.4) | 2.35 (1.22, 4.52) |  |
| Synovial hypertrophy and ROA | 48 (73.9) | 8.20 (3.28, 20.52) |  |
| **Joint effusion** |  |  | 0.033 |
| Neither joint effusion nor ROA | 92 (43.6) | 1.00 (reference) |  |
| Joint effusion only | 41 (44.6) | 1.23 (0.66, 2.30) |  |
| ROA only | 47 (50.0) | 2.06 (1.02, 4.18) |  |
| Joint effusion and ROA | 66 (69.5) | 7.83 (3.29, 18.66) |  |
| **Power Doppler signal** |  |  | 0.961 |
| Neither Power Doppler signal nor ROA | 130 (43.5) | 1.00 (reference) |  |
| Power Doppler signal only | 3 (75.0) | 5.81 (0.55, 61.92) |  |
| ROA only | 96 (57.5) | 3.17 (1.69, 5.97) |  |
| Power Doppler signal and ROA | 17 (77.3) | 9.33 (2.47, 35.24) |  |

OR, odds ratio; CI, confidence interval; ROA, radiographic osteoarthritis.

* adjusted for knee injury history.
